# Continuous Glucose Monitoring Versus Self-monitoring of Blood Glucose in Type 2 Diabetes Mellitus: A Systematic Review with Meta-analysis

**DOI:** 10.7759/cureus.5634

**Published:** 2019-09-12

**Authors:** Rajesh Naidu Janapala, Joseph S Jayaraj, Nida Fathima, Tooba Kashif, Norina Usman, Amulya Dasari, Nusrat Jahan, Issac Sachmechi

**Affiliations:** 1 Internal Medicine, Icahn School of Medicine at Mount Sinai/Queens Hospital Center, New York, USA; 2 Internal Medicine, California Institute of Behavioral Neurosciences and Psychology, Fairfield, USA; 3 Internal Medicine, Veterans Affairs Palo Alto Health Care System - Stanford University School of Medicine, Palo Alto, USA

**Keywords:** continuous glucose monitor, type 2 diabetes mellitus, self-monitoring of blood glucose, real-time glucose monitoring, time in range, glucose variability

## Abstract

Every eleventh adult has diabetes, and every third has prediabetes. Over 95% of diabetics are of type 2. It is well established that diabetes doubles the risk of heart disease and stroke apart from increasing the risk of microvascular complications. Hence, strict glycemic control is necessary. However, it increases the risk of hypoglycemia, especially in patients with longstanding diabetes. Continuous glucose monitors (CGM) use a sensor to continuously measure the glucose levels in the interstitial fluid every 10 seconds and gives out mean values every five minutes. CGMs are emerging tools in the management of type 2 diabetes. The prime objective of this review is to find out if there is enough supporting evidence, suggesting that continuous glucose monitoring is more effective than self-monitoring of blood glucose (SMBG) in type 2 diabetes. We conducted a systematic literature search in Medline (PubMed) looking for any studies addressing our objective. It is observed that there is a varying level of evidence supporting that employing a CGM can reduce glycated hemoglobin (HbA1c), hypoglycemic events, and increase patient satisfaction. However, some studies reported no significant benefits. This systematic review with meta-analysis concludes that the use of CGM in type 2 diabetes mellitus (T2DM) is beneficial, as it significantly reduces HbA1c compared to the usual method of SMBG. The pooled mean difference in HbA1c was -0.25 (-0.45, -0.06) and statistically significant (at *p* = 0.01) when comparing CGM to SMBG.

## Introduction

The International Diabetes Federation estimates that one in every 11 adults has diabetes accounting for about 425 million diabetics in the world [1]. While the Center for Disease Control and Prevention states that the United States alone has 30 million diabetics (of whom 95% are type 2 diabetics) and 90 million prediabetics [2]. Apart from being the leading cause of chronic kidney disease, lower-limb amputations, and adult-onset blindness, diabetes also doubles the risk of having heart disease or stroke. Diabetes is the seventh leading cause of death in the United States. The financial burden of diagnosed diabetes is projected as $327 billion yearly, which is going to increase exponentially as the population is aging and living longer than before.

The Benefits of Continuous glucose monitor (CGM) in type one diabetic (T1DM) patients when compared to routine glucose testing have been very well established by many studies and are now a vital tool in their diabetes management. However, the benefits of CGM in type 2 diabetics (T2DM) are not well established, and its usage is limited. As most of the T2DM patients are elderly, their diabetes management is a challenge due to the co-existence of multiple comorbidities and polypharmacy. Although glycated hemoglobin (HbA1c) is a gold standard marker to assess glycemic control and a well-established marker correlating with increased complications, CGM gives the power to make the diabetes management personalized [3-4]. Though it is well demonstrated that bringing down HbA1c to <7% by an intensive glycemic control decreases the risk of microvascular complications, it is associated with an increased risk of hypoglycemia [5]. With increasing accuracy and features such as hypoglycemia alarms and trends, CGMs can reduce the risk of severe hypoglycemic events. Through this review, we wanted to know if there is enough evidence to support the efficacy of CMG over self-monitoring of blood glucose (SMBG) in patients with T2DM.

In this article, we discussed what is already known, not known, and the emerging trends in the usage of CGM in patients with T2DM. We also discussed the emerging parameters of blood glucose measurements that will potentially replace HbA1c in guiding treatment decisions. We conducted a meta-analysis compiling data from different studies to know if CGM usage can effectively reduce HbA1c in T2DM patients.

## Materials and methods

**Figure 1:**
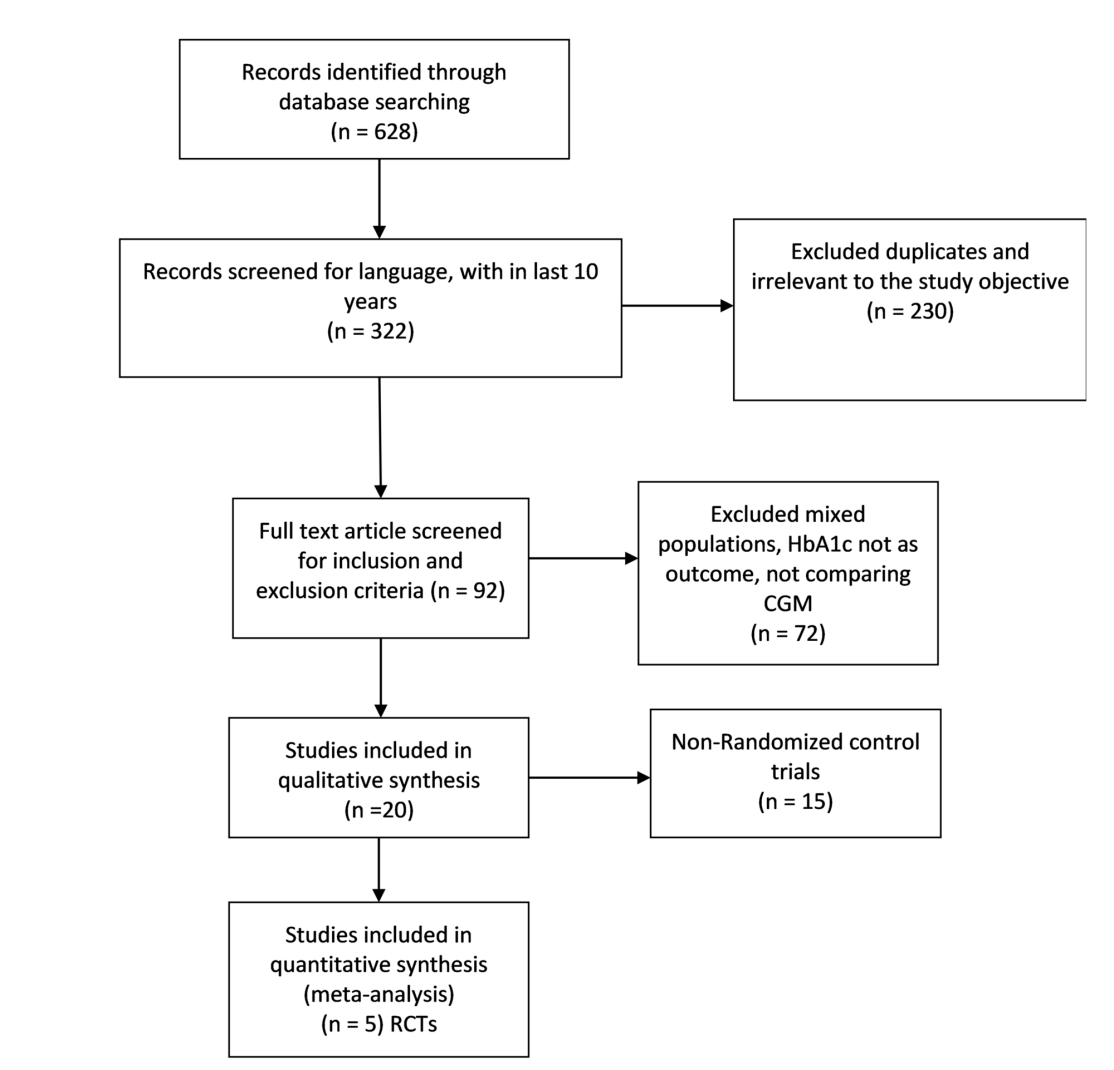
Summary of study flow (PRISMA flow diagram) PRISMA = preferred reporting items for systematic reviews and meta-analyses, RCT = randomised controlled trial, n = number of results

In this trial, we conducted a thorough and systematic literature search in MEDLINE (PubMed) through a combination of both Mesh terms and keywords. The following table details the search strategy (Table 1). The search terms were separately developed by two authors individually and combined to perform a comprehensive search of relevant literature from the last 10 years. Studies were screened for inclusion and exclusion criteria, as mentioned below. The following figure (Figure 1) summarizes the flow of search trial through the Preferred Reporting Items for Systematic Reviews and Meta-Analyses (PRISMA) flow diagram [6].

**Table 1:** Search words and their combined results MeSh, medical subject headings

Population(P):	Article hits
"Diabetes Mellitus"[Mesh] OR "Diabetes Mellitus, Type 2"[Mesh]	405131
AND	
Intervention(I):	
(("continuous glucose monitoring"[All Fields] OR "CGM"[All Fields]) OR "real-time glucose monitoring"[All Fields]) OR (continuous [All Fields] AND "measurement"[All Fields]) AND ("glucose"[MeSH Terms] OR "glucose"[All Fields])	4406
AND	
Comparison(C):	
(("Blood Glucose Self-Monitoring"[Mesh] OR "self glucose monitoring"[All Fields]) OR (intermittent[All Fields] AND ("blood glucose self-monitoring"[MeSH Terms] OR ("blood"[All Fields] AND "glucose"[All Fields] AND "self-monitoring"[All Fields]) OR ("blood glucose self-monitoring"[All Fields]) OR ("self"[All Fields] AND "blood"[All Fields] AND "glucose"[All Fields] AND "monitoring"[All Fields]) OR ("self blood glucose monitoring"[All Fields])) OR "Home glucose monitoring"[All Fields]	6313
AND	
Outcomes(O):	
((((((((("Glycated Hemoglobin A"[Mesh] OR "hemoglobin A1c"[All Fields]) OR "HbA1c"[All Fields]) OR "Hypoglycemia"[Mesh]) OR "Hypoglycemic episodes"[All Fields]) OR "Hypoglycemic episode"[All Fields]) OR "low blood glucose"[All Fields]) OR "ease of use"[All Fields]) OR "convenient"[All Fields]) OR "convenience"[All Fields]) OR "user-friendly"[All Fields]	175204
Final Search results:	628

Inclusion

1. Studies that compare CGM of blood glucose to SMBG (or other routine methods for monitoring hyperglycemia) in T2DM patient (≥19 years of age)

2. Studies that measure HbA1c as an outcome and has a baseline mean HbA1c ≥6.5%

3. Articles that are in English language or other languages if a translated version in English is readily available.

Exclusion

1. Studies involving pregnant women

2. In-patient population

Statistical analysis

The statistical analysis is planned to be carried out with Review Manage 5.3 (RevMan 5.3). The primary outcome to be measured in this review is the difference of mean HbA1c in the CGM group compared to the SMBG group at the end of the studies. In a randomized control trial, it is assumed that difference in final mean measurements will on average be the identical estimate of the difference in mean change measurements. Heterogeneity will be determined by I² static. I² 50% or more is regarded as substantial heterogeneity among the studies. A fixed-effect model will be used to combine the individual study results if heterogeneity is low (<20 %) or else the random-effects model will be used.

## Results

Data extraction and quality appraisal

Data extracted from the randomised control trials (RCT) from our literature search are shown in Table 2.

**Table 2:** Data extracted from the RCTs n = total number of subjects, I = number of subjects in the intervention group (CGM group), C = number of subjects in the control group (SMBG group), RCT = randomised control trial [7-11]

	First author/Year of publication	Study population	Study Duration	CGM usage duration	Outcomes compared to control group			Limitations
					HbA1c	Hypoglycemia	Ease of use/ Quality of life	
1	Beck RW et al., 2017	n=158 I=79 C=79	24 Weeks/six months	Daily usage for 24 weeks	The adjusted difference in mean change for CGM group and control group is -0.3% [95% CI, -0.5% to 0.0%]; P = 0.022)	Did not differ meaningfully in measured hypoglycemia	Did not differ meaningfully in Quality of life measures. However, the CGM group had high satisfaction with use of CGM	Study duration. CGM-measured hypoglycemia was extremely low at baseline, which limited the ability to assess the effect of CGM on reducing hypoglycemia.
2	Yoo HJ et al., 2008	n = 65 I = 32 C = 33	12 weeks/ three months	Monthly three days at a time for three months	Significantly reduced (9.1 ± 1.0% to 8.0 ± 1.2% vs. 8.7 ± 0.7% to 8.3 ± 1.1%, respectively; P = 0.004)	No significant difference between the groups	Significant reduction in total daily calorie intake, weight, body mass index (BMI), and postprandial glucose level, and a significant increase in total exercise time per week.	Small study population and short study duration
3	Ehrhardt NM et al., 2011 and Vigersky RA et al., 2012	n = 100 I = 50 C = 50	12 weeks of intervention and 52-week long term follow up	12 weeks of intermittent usage	Significant decrease in mean, unadjusted HbA1c at end of 12 weeks of intermittent CGM usage (1.0% vs0.5%) and sustained at week 40(0.8%vs 0.2%) (P = 0.04). Average, statistically adjusted Decline of -0.48% (p = .006)	Not assessed	No difference in Weight, Blood pressure, and The Problem Areas in Diabetes (PAID) scores.	Small study population, a slight variation in baseline characteristics (age)
4	Sato J et al., 2016	n = 34 I = 17 C = 17	Eight months	Four to five days of usage on three separate occasions	No significant difference in the change of HbA1c at the end of the study	Time spent in hypoglycemia was almost zero in both groups both at baseline and at the end of the study. Hence the difference between the groups was not appreciated.	Based on changes in Diabetes Treatment Satisfaction Questionnaire (DTSQ) scores, No significant improvement in patient satisfaction.	small sample size
5	Cosson E et al., 2009	n = 25 I = 11 C = 14	three months	48 hrs.	significantly reduced (mean: –0.63±0.34%; P= 0.05 vs –0.31±0.29%; P= 0.18, respectively)	No significant difference between the groups	Most patients reported no or mild pain, while mixed reporting on bothersome of the device due to its bulkiness.	Small study population and short study duration

**Figure 2:**
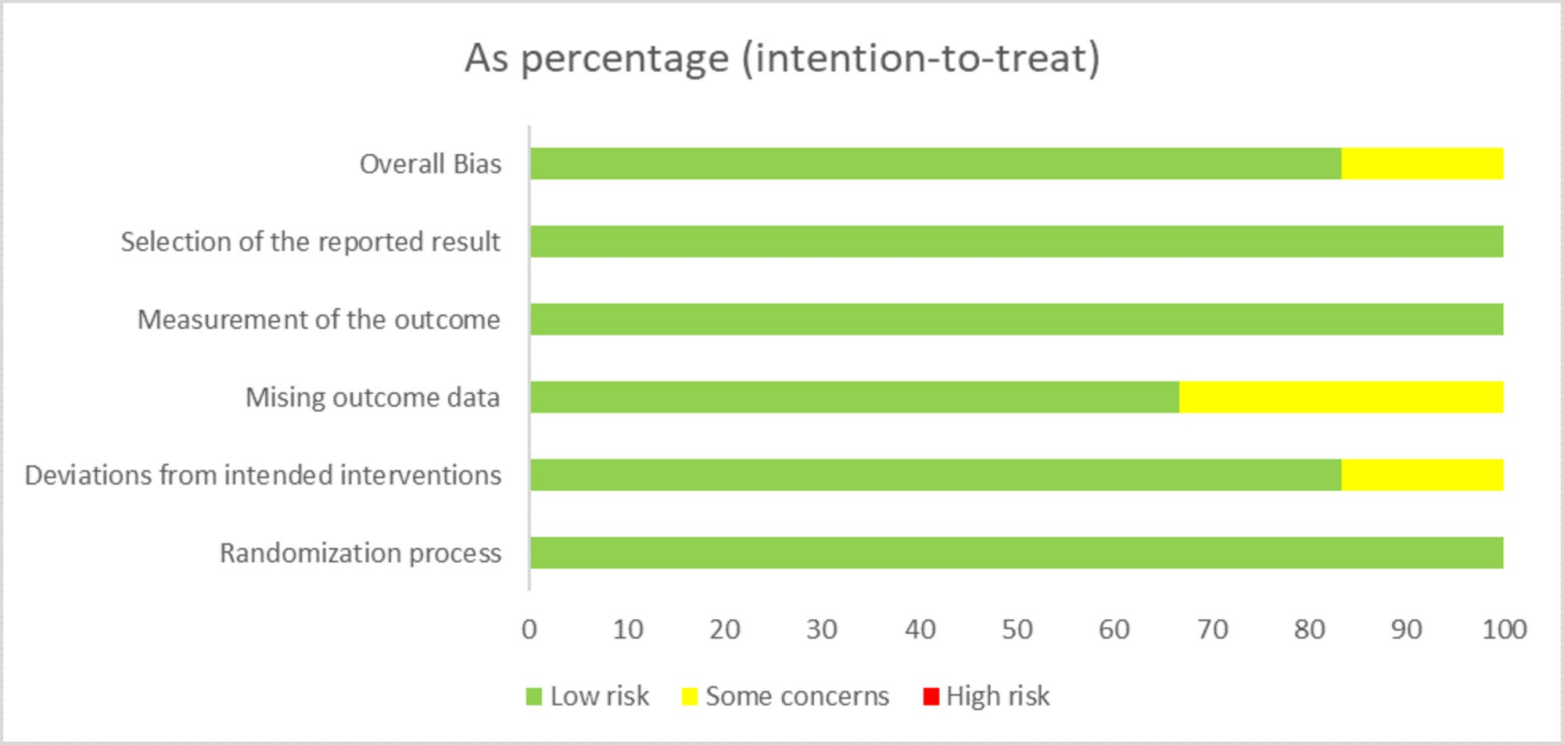
Illustrative summary of RoB of the RCTs RoB = Risk-of-bias, RCT = randomized control trials

Quality assessment was done in duplicate by two authors independently for all the RCTs using the latest revised Cochrane Risk-of-Bias (RoB) tool for randomized trials [13]. The summary of the risk of bias for the six randomized trials is shown illustratively in Figure 2. During the quality assessment, any discrepancy between the authors was solved after discussion with a third author. Five of the six studies shown to have a low risk of bias, while one study proved to have some concerns [7-12]. A systemic review from our search was assessed for quality with the AMSTAR checklist [14]. All other studies were evaluated for their quality using study type-specific Critical Appraisal Checklist from Joanna Briggs Institute (JBI) [15]. Each questionnaire has 10-11 questions. Each question was given one point. A study scoring five or fewer points was considered having a high risk of bias.

All the studies were thoroughly comprehended, and their relevant historical bibliographic references were searched for all pertinent information. After excluding studies that have data only from T1DM patients and including studies that have data from only T2DM patients or mixed population (but with subgroup data and analysis for T2DM), resulted in six RCTs. Two of these studies reported data for the same study at different time points (one after the intervention and another one after long term follow-up without intervention) [9-10]. Hence, only five studies were included in the meta-analysis.

The five RCTs studied 382 T2DM patients that met our study criteria with 189 patients being in the CGM group and 193 in the control group. However, in a study, three subjects in the intervention group and five subjects in the control group dropped out and were not included in that studies’ final analysis [8]. Hence, our meta-analysis has 374 total T2DM patients, which include 186 in the CGM group and 188 in the SMBG group. The studies lasted in a range of three to eight months in duration. The baseline HbA1c levels ranged between 6.9% to 12%. Moreover, the cumulative mean HbA1c for all the five RCTs was 8.53% (0.91) at baseline, indicating poorly controlled diabetes.

Meta-analysis

**Figure 3:**
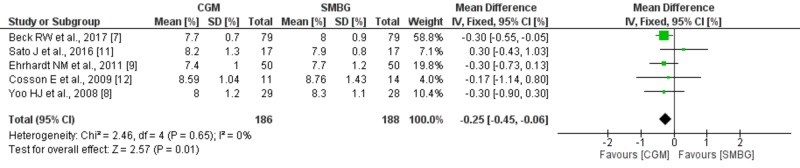
Mean difference of HbA1c between CGM and SMBG groups at the end of respective studies and their pooled analysis CGM = continuous glucose monitoring, SMBG = self-monitoring of blood glucose, SD = standard deviation, CI = confidence interval

**Figure 4:**
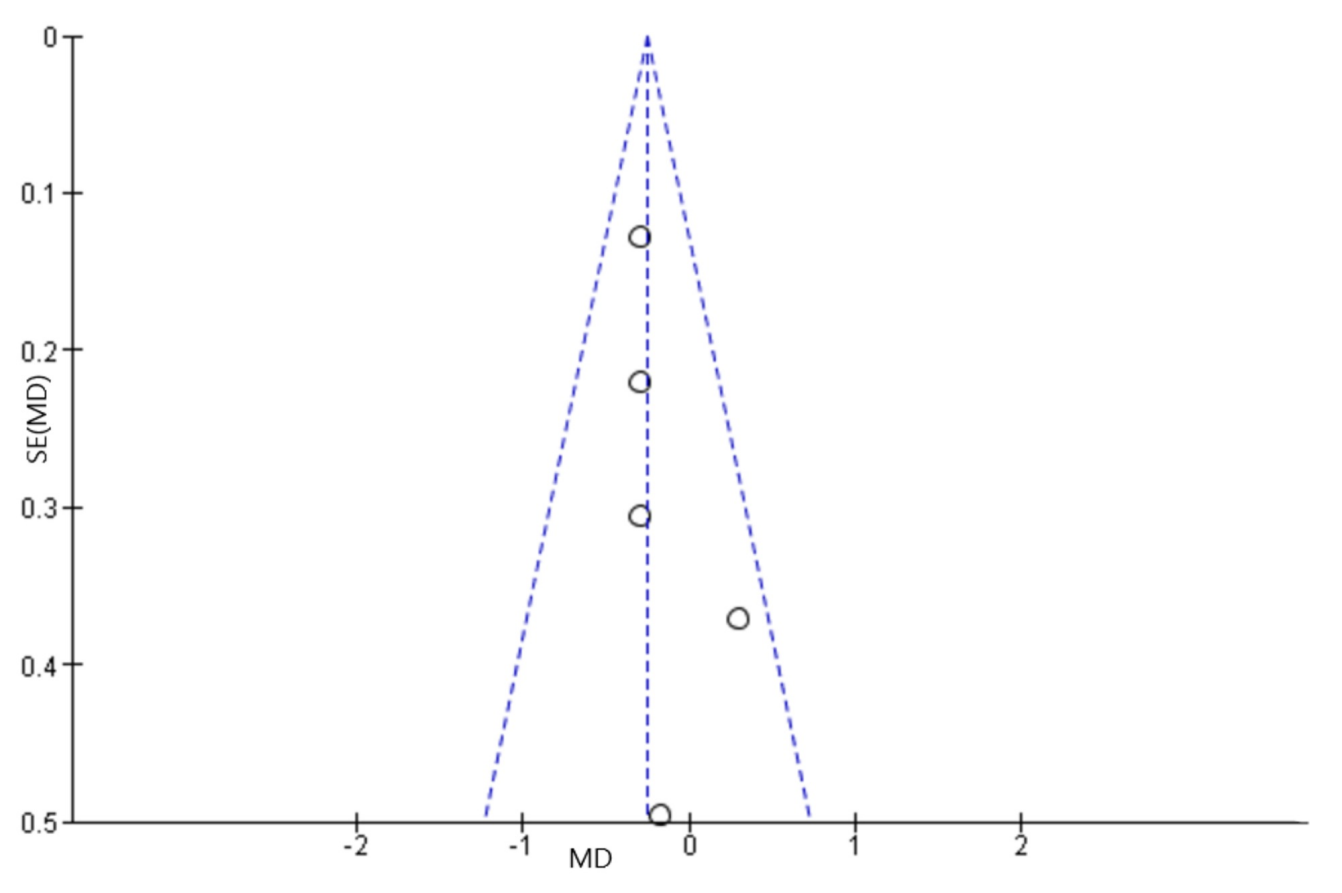
Funnel plot for the five RCTs comparing CGM to SMBG in T2DM MD = mean difference, SE = standard error, RCTs = randomized control trials, CGM = continuous glucose monitor, SMBG = self-monitoring of blood glucose, T2DM = type 2 diabetes mellitus

Cumulative analysis of the data from all five RCTs was done using RevMan 5 tool. The fixed-effect model was used to combine the results as the heterogeneity was very low (I² = 0%), suggesting minimal variation across studies. The cumulative analysis of data from all the five RCTs showed that CGM usage in T2DM patients decreased HbA1c by 0.25% (with a 95% confidence interval between 0.45 and 0.06) compared to SMBG (*p* = 0.01). The pooled mean difference in Hba1c was -0.25 (-0.45, -0.06) with statistical significance of (*p* = 0.01) comparing CGM to SMBG. A forest plot illustrating the same is shown in the figure (Figure 3). The funnel plot (Figure 4) shows that no publication bias was observed.

## Discussion

CGMs are the latest tools in the management of diabetes. The first CGM was approved by the Food and Drug Administration in 1999. Since then they kept evolving from a large device with physical wires to the sensor to present-days virtually painless small sensors that communicate wirelessly with their receivers. Current commercially available CGMs comes with different features and functions, but the main idea is that they have a sensor that is usually attached to the skin either over the abdomen or back of the arms. The tip of the sensor lies in the interstitial fluid measuring interstitial glucose level every ten seconds, then giving out an average reading every five minutes and up to 288 readings in a day, which is then transmitted to the receiver or smartphone wirelessly.

This review discusses all the aspects of CGM in type the diabetics that we came through our literature review. The CGM group or intervention group was compared to a control group that was using either an SMBG with multiple finger sticks or other routine methods. Most studies emphasized on the following areas when measuring the outcome of using CGM in T2DM patients. These include HbA1c, hypoglycemia, glucose variability, and patient satisfaction.

HbA1c

Change in the HbA1c was the primary outcome in most of the studies that focused on CGM usage in T2DM. Most studies have demonstrated a reduction in HbA1c with the use of CGM when compared to the controls [7-10,13,16,17]. However, a study done in a university hospital in Japan and published in 2016 demonstrated no significant change in HbA1c when compared to control [11]. It is noted that the study sample size was very small compared to other studies. In our meta-analysis, the analysis of pooled data from five RCTs showed that CGM was more effective in reducing HbA1c (mean difference of -0.25 and 95% confidence interval between -0.45and -0.06) compared to SMBG with a statistical significance of more than 95% (*p* = 0.01). The cumulative mean HbA1c of the five RCTs was 8.53% (0.91) at the baseline, indicates that CGM was effective in T2DM patients with poorly controlled diabetes. It was observed that 14 days of CGM usage provided a reasonable estimate of mean glucose, time in range, and hyperglycemia measures for three months [18]. Collecting data for additional days did not prove to improve correlation to standard glucose metrics like HbA1c and mean glucose. Twelve weeks of intermittent usage of Real Time-CGM (RT-CGM) has shown not only to reduce HbA1c but also the effect was sustained at week 40, even after the discontinuation of CGM at 12 weeks [10]. This demonstrated that even short-term usage of CGM was beneficial, which might be due to the constant feedback of diet-related glucose variations resulting in patients becoming more aware of what foods to choose. This feedback mechanism leads to healthy lifestyle modification, which is very much needed in patients with T2DM.

Hypoglycemia 

Hypoglycemic episodes could become a prime indication for choosing CGM by care providers in the management of their patient’s diabetes. Tighter glycemic control is limited by events of Hypoglycemia. Long-standing diabetes leads to peripheral neuropathy and can reduce hypoglycemic awareness in the elderly. It is observed that in the elderly with diabetes, hypoglycemia is a more common cause for hospitalization than hyperglycemia [19]. It also increases the risk of mortality.

Studies demonstrated that CGMs detected a significantly higher number of hypoglycemic events than the SMBG or symptomatic hypoglycemia [20-23]. It is noticed that the hypoglycemic episodes were predominantly nocturnal [17,21,22,24]. Hence, naturally, they will go unnoticed by the patient or patient’s partner, which makes the timely rescue challenging and increases the risk of mortality. CGMs with alarms to extreme glycemic excursions help in detecting impending severe hypoglycemic episodes and helps take timely action. However, it is noted that some studies have shown that CGM data did not differ significantly from the controls], which may be explained by the fact that these populations could be relatively healthy with lesser glycemic excursions [7,10,12]. Therefore, these studies have insufficient power in detecting a significant difference between the groups.

Glucose variability and time in range

HbA1c and SMBG can accurately estimate the mean glucose values, but they lack the power to look for glycemic excursions. It is observed that most hyperglycemia occurs post postprandially while most hypoglycemia occurs during the night, which is missed by T2DM patients who routinely do finger pricks in the morning and before meals. On the other hand, HbA1c only correlates with mean blood glucose levels leaving out extreme values. Additionally, studies have reported that obesity could falsely show low HbA1c [25].

Extensive glycemic data yielded by the CGMs can help overcome the above limitations. They can report the time spent in specific glycemic ranges in a day. That is, they can report the amount of time spent with glycemic levels ≤70 mg/dl, between 70 and 180mg/dl and ≥180 mg/dl. Even custom glycemic ranges can be set to personalize the management. The emerging new parameter in diabetes management is Time in Range (TIR). Studies have demonstrated that CGM usage helped in increasing the time spent in TIR and decrease in time spent in hypoglycemia and hyperglycemia [7,10,26]. As of now, there is no standardized range for TIR, but the most acceptable range is between 70 and 180 mg/dl below 70 mg/dl is regarded as hypoglycemia and above 180 mg/dl as hyperglycemia. Efforts are being made to standardize the CGM metrics, including TIR, in establishing the goals for diabetes management. American Diabetic Association has presented recommendations for TIR in Type 1 and 2 diabetics as between 70 and 180 mg/dl in June 2019 [27].

Patient satisfaction

Patient satisfaction is one of the critical aspects that decide if the CGM can be used in daily life. Unlike the HbA1c (which is done once in three months) or SMBG (which is performed once or twice daily in most type 2 diabetics), a CGM device is attached to a patient’s body throughout the day for 7 to 14 days or more. Unless the patients are satisfied with the accuracy, usability, and the benefits of the device, they might not be motivated to wear them. Surprisingly, one study reported very high compliance with CGM usage; about 97% of the subjects used it for six or more days per week for six months. A satisfaction survey at the end of the trial indicated very high satisfaction with CGM [26]. Some other studies also demonstrated similarly high satisfaction [7,28].

One study observed that there was a significant reduction in daily calorie intake, body mass index, postprandial glucose levels, and increased exercise time per week in patients using CGM for three consecutive days within three months [10]. Real-time glucose data might serve as a motivational tool for patients, as they receive real-time feedback on their diet and exercise, encouraging them to adopt a healthy lifestyle in the long term. However, some studies reported no significant difference in weight, blood pressure, Problem Areas in Diabetes (PAID) scores, or patient satisfaction [8-9,11].

Personal and professional CGM devices

CGM devices can be classified as professional CGM devices and personal CGM devices. Personal CGM devices display real-time glucose measurements to patients. Which helps patients understand the effects of diet and lifestyle on their blood glucose levels. CGMs can act as a motivational tool and also guide medication dosage in patients using insulin. They also alert patients to extreme glucose excursions. Professional CGM devices are blinded to patients. They provide retrospective data which is mainly used by healthcare providers in making necessary changes to the patient’s diabetes regimen. They are worn by the patient generally for a period of seven days, and then the data is downloaded by the healthcare provider. Professional CGM has demonstrated that it improves both glycemic control and cost outcomes in patients over a wide range of baseline therapies [17,29]. One study noticed that professional CGM devices provided the maximal benefit for patients with a baseline HbA1c level of 7% or above [17]. Currently, CGM devices are expensive and are not covered by most insurance providers for T2DM. However, professional CGM devices can be cost-effective and user-friendly. Thus, they can be potentially incorporated into primary care.

Limitations of CGM devices

Like any other tool in managing diabetes CGM devices also suffer from some limitations. Currently, the most limiting factor for its integration into routine diabetic care is the cost. CGM devices are expensive and prescription only. The initial cost may exceed over $1000 for the device, and a monthly supply of sensors may cost over $300. Some of the devices need a fingerstick glucose test done to calibrate the CGM device frequently. This may be annoying for patients as they cannot rely on the CGM data itself, especially with extreme readings. However, the current generation of CGM devices are improving in accuracy and are factory calibrated; hence, they do not need end-user calibration. One of the prime reasons for adopting CGM devices for some patients is to manage their hypoglycemia. However, it is to be noted that CGM devices show increasingly inaccurate results at low glucose ranges. Unlike finger stick tests where the glucose levels are measured in capillary blood, CGM devices measure it in the interstitial fluid. There is a time lag of 5 to 20 minutes before the vascular and interstitial glucose levels equilibrate. Hence, they can be unreliable at times, especially during rapid fluctuations. Some people may feel uncomfortable to wear a device that is stuck on their skin all the time. Lastly, Personal CGM devices might not be beneficial merely by wearing them, without any insight and motivation to make lifestyle modifications in line with the feedback from the real-time glycemic data.

Limitations of this review

This review has many limitations. Importantly, the literature search was conducted only in one electronic database, Medline (PubMed) database. Some relevant and critical studies that are not PubMed indexed might have been missed. Studies published in other than the English language (except if their translated version is readily available) have not been reviewed. No detailed sensitivity analysis is performed due to the small sample size of the studies. No explicit cost-benefit or burden has been studied in this review.

## Conclusions

The benefits of CGM in T1DM patients have been well established. However, relatively few studies have been conducted in T2DM patients, and most of them are of less than six months in duration. Although a significant amount of evidence suggests that the usage of CGM in the management of T2DM is associated with benefits of reduction in HbA1c (from our meta-analysis) especially in poorly controlled T2DM patients, the sample size and study durations are too small to generalize the results. We see the need for RCTs with larger sample sizes and longer durations to establish the above beneficial effects. The metrics of CGM have to be standardized so that they can be widely adopted into clinical practice. Guidelines for the indication of a CGM in T2DM have to be established.
